# Beninese Medicinal Plants as a Source of Antimycobacterial Agents: Bioguided Fractionation and *In Vitro* Activity of Alkaloids Isolated from *Holarrhena floribunda* Used in Traditional Treatment of Buruli Ulcer

**DOI:** 10.1155/2015/835767

**Published:** 2015-05-28

**Authors:** Achille Yemoa, Joachim Gbenou, Dissou Affolabi, Mansourou Moudachirou, André Bigot, Séverin Anagonou, Françoise Portaels, Anandi Martin, Joëlle Quetin-Leclercq

**Affiliations:** ^1^Unité de Formation et de Recherche en Pharmacie, Faculté des Sciences de la Santé (FSS), Université d'Abomey Calavi (UAC), 04 BP 494 Cotonou, Benin; ^2^Laboratoire de Pharmacognosie et des Huiles Essentielles (LAPHE), Faculté des Sciences de la Santé (FSS) and Faculté des Sciences et Techniques (FAST), Université d'Abomey Calavi (UAC), 01 BP 188 Cotonou, Benin; ^3^Laboratoire de Référence des Mycobactéries (LRM), Centre National Hospitalier de Pneumo-Phtisiologie (CNHPP), 01 BP 817 Cotonou, Benin; ^4^Department of Biomedical Sciences, Institute of Tropical Medicine (IMT), Nationalestraat 155, 2000 Antwerpen, Belgium; ^5^Laboratory of Microbiology, Department of Biochemistry and Microbiology, Faculty of Sciences, Ghent University, K.L. Ledeganckstraat 35, 9000 Gent, Belgium; ^6^Pharmacognosy Research Group, Louvain Drug Research Institute (LDRI), Université Catholique de Louvain (UCL), B1 7203 Avenue E. Mounier 72, 1200 Bruxelles, Belgium

## Abstract

Buruli ulcer (BU) imposes a serious economic burden on affected households and on health systems that are involved in diagnosing the disease and treating patients. Research is needed to find cost-effective therapies for this costly disease. Plants have always been an important source of new pharmacologically active molecules. Consequently we decided to undertake the study of plants used in traditional treatment of BU in Benin and investigate their antimycobacterial activity as well as their chemical composition. Extracts from forty-four (44) plant species were selected on account of reported traditional uses for the treatment of BU in Benin and were assayed for antimycobacterial activities. Crude hydroethanolic extract from aerial parts of *Holarrhena floribunda* (G. Don) T. Durand and Schinz was found to have significant antimycobacterial activity against *M*. *ulcerans* (MIC = 125 *µ*g/mL). We describe here the identification of four steroidal alkaloids from *Mycobacterium ulcerans* growth-inhibiting fractions of the alkaloidal extract of the aerial parts of *Holarrhena floribunda*. Holadysamine was purified in sufficient amount to allow the determination of its MCI (=50 *µ*g/mL). These results give some support to the use of this plant in traditional medicine.

## 1. Introduction

Buruli ulcer (BU), caused by the environmental organism* Mycobacterium ulcerans* and characterized by necrotizing skin and bone lesions, poses important public health issues as the third most common mycobacterial infection in humans [1]. The disease has become substantially more frequent over the past decade, particularly around the Gulf of Guinea, and has been detected or suspected in at least 31 countries. Clinical diagnosis of BU disease should be confirmed by PCR, as recommended by the World Health Organization (WHO), and case patients should be treated with rifampin/streptomycin daily for 8 weeks (therapy available since 2004), combined, if necessary, with surgery.

In Benin, sociocultural believes and practices strongly influence the health-seeking behaviours of people affected by BU. The first recourse is often traditional treatment. Most of the components in the traditional treatment belong to the plant kingdom [2]. Plants provide unlimited opportunities for new drug leads because of the unmatched availability of chemical diversity. Recently we carried out an ethnobotanical survey involving seventeen traditional practitioners within the Ouinhi community in the Zou Department (Benin). We noted that about forty-nine different plants were used for the traditional treatment of BU. Different parts of these plants were included in various pharmaceutical forms for internal or external use [2]. We realized a screening of 44 plant extracts used in traditional medicine to treat BU [2, 3]. Results showed that crude hydroethanolic extract of* Holarrhena floribunda* was effective in inhibiting the growth of* Mycobacterium ulcerans *(MIC 125 *μ*g/mL) and worth further investigations.

**Figure 1:**
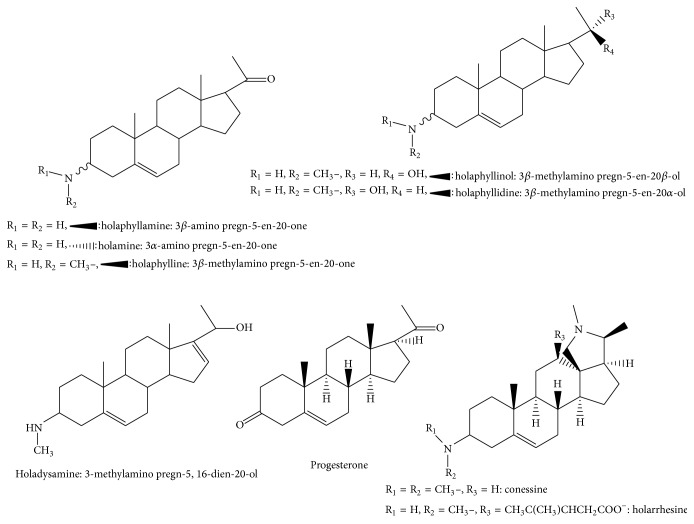
Structures of chemical compounds isolated from* H. floribunda*.

*Holarrhena floribunda *(G. Don) T. Durand and Schinz grows as a shrub or tree up to 25 m tall, with a stem diameter of up to 30 cm. Its fragrant flowers feature a white corolla. Fruit is pale grey to dark brown with paired follicles, each up to 60 cm long. This species is known as four synonyms:* Rondeletia floribunda *G. Don,* Holarrhena africana* A. DC.,* Holarrhena wulfsbergii* Stapf,* Holarrhena ovata. *In the Republic of Benin, the common names of this plant are: Fon: “kpakpatoun” [2]; Yoruba and Nago: “ako ire,” “ire Ibedji;” Mina: “gaoti” [4]. Hoyer et al. [5] in 1978 isolated from the bark of the trunk a steroidal alkaloid called holarrhesine, but no activity was reported for this compound. They also isolated conessine from the bark of the root. A chemical study of the leaves of* H. floribunda* achieved by Janot team in 1959 led to the isolation of other new alkaloids including holaphylline and holaphyllamine, while other alkaloids were isolated later.  Figure 1 shows the structures of the 9 chemical compounds isolated from* H. floribunda*: holaphylline, holaphyllamine, holamine, holaphyllinol, holaphyllidine, holadysamine, holarrhesine, conessine, and progesterone [6–9]. Phytochemical investigations on this plant have so far led to the identification of a crude* in vitro* active alkaloid extract on* M. tuberculosis* (MCI = 0.075 g/L) and other microorganisms [10, 11].

Here we describe compounds identified from bioactive fractions of the extract and evaluation of the inhibitory effect of one of them on the growth of* M. ulcerans*.

## 2. Materials and Methods

### 2.1. Experimental

#### 2.1.1. Plant Materials

Plant species was collected and identified by a botanist from the National Herbarium of Benin and voucher specimens (Yemoa 06) are deposited at the same herbarium.

#### 2.1.2. Preparation of Crude Hydroethanolic Extracts

Dried plants were ground to a powder with a pulverizator (National Mixer Grinder Mx-119N, Japan). 50 g of powder was then macerated 48 h (at room temperature) in 70% ethanol in a 1/10 (w/v) ratio. The material was filtered through a Millipore filter of 0.2 *μ*m (Acrodisc, USA). The filtrate was concentrated under reduced pressure at less than 40°C using a rotary evaporator (Buchi Rotavapor R-200/205, Switzerland) to obtain a crude residue.

#### 2.1.3. Fractionation of Crude Extracts and Isolation of Active Constituents

**Figure 2:**
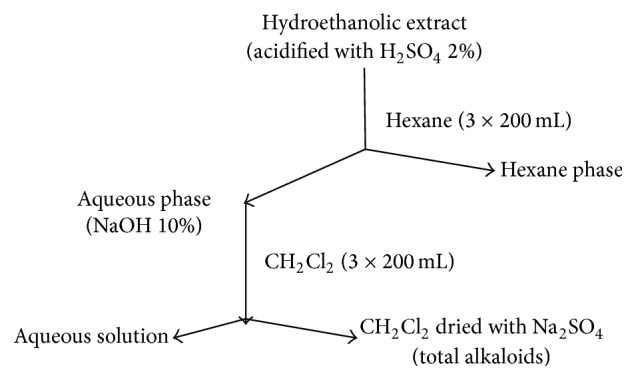
Scheme for preparation of alkaloids enriched extract.

Crude hydroethanolic extract was fractionated on silica gel 60 (0,063–0,200 mm Merck, Germany) by atmospheric pressure liquid chromatography eluting with solvents of increasing polarity, namely, hexane, dichloromethane, ethyl acetate, and water, yielding 4 fractions (F1, F2, F3, and F4). Of these, fraction F2 (dichloromethane) was found to cause growth inhibition of* M. ulcerans *and was, as a result, selected for further work. This fraction was monitored using TLC plates (TLC silica gel 60F_254S_, Merck) and led to characterize the presence of alkaloids (toluene : ethyl acetate : diethylamine: [7 : 2 : 1], detection-Dragendorff's spray reagent). We then performed a more specific extraction to obtain an enriched alkaloid extract (Figure 2).

We therefore proceeded to fractionation of this enriched extract by Atmospheric Pressure Liquid Chromatography (APLC) and Medium Pressure Liquid Chromatography (MPLC) followed by gel filtration on Sephadex LH20 to purify this enriched alkaloid fraction.

*Atmospheric Pressure Liquid Chromatography (APLC).* Alkaloids enriched extract was repeatedly fractionated on silica gel 60 (0,063–0,200 mm, Merck, Germany) eluting with solvents of increasing polarity (mixture of CH_2_Cl_2_/MeOH 50 : 50 to 100% MeOH followed by MeOH : H_2_O 90 : 10 and MeOH : acetic acid 90 : 10). Fractions were monitored by TLC (toluene : ethyl acetate : diethylamine :  [7 : 2 : 1], detection-Dragendorff's spray reagent) and similar fractions were combined and concentrated in vacuo. Prepurified fractions obtained by APLC were rechromatographied by MPLC.

*Medium Pressure Liquid Chromatography (MPLC).* MPLC was performed on glass columns packed with LiChroprep Si 60 (15–25 *μ*m) from Merck with a mobile phase composed of CH_2_Cl_2_/MeOH 50 : 50 to 100% MeOH. Prepurified fractions obtained were monitored by TLC (toluene : ethyl acetate : diethylamine: [7 : 2 : 1], detection-Dragendorff's spray reagent) and similar fractions were combined and concentrated in vacuo followed by gel filtration on Sephadex LH20 (MeOH). Five different purified fractions were obtained and analyzed by high pressure liquid chromatography coupled to mass spectroscopy (HPLC-MS or LC-MS) (Thermo Scientific Accela LC Systems, orbitrap).

*LC-MS.* High pressure liquid chromatography coupled to a diode array detection and mass spectrometry with positive electrospray ionization (HPLC-ESI-MS^n^) was employed to rapidly separate and identify the constituents in these five purified fractions. The LC MS/MS system consisted in a Thermo Accela pump, autosampler, photodiode array detector, and Thermo Scientific LTQ orbitrap XL mass spectrometer.

Separation was performed using an analytical RP-C18 Lichrospher-100 column (250 × 4 mm, particle size 5 *μ*m) with a gradient using acetonitrile and 0, 05% trifluoroacetic acid (TFA) aqueous solution as the mobile phase. The gradient used starts at 90% aqueous solution and 10% acetonitrile, going to a plateau of 100% acetonitrile in 25 min. These conditions are held for 15 min before returning to the initial conditions. The gradient was linear and the flow rate was 0.4 mL/min. The injection volume was 10 *μ*L; the column temperature was 30°C.

High-resolution MS was measured with ESI source in the positive mode. The following inlet conditions were applied: capillary temperature 275°C, capillary voltage 35 V, tube lens 110 V, sheath gas flow 8 u.a, auxiliary gas flow 0 u.a, and sweep gas flow 0 u.a. Data acquisition and processing were performed with Xcalibur software version 2.0.7.

*NMR.* NMR spectra (^1^H, ^13^C, ^1^H-^1^H COSY, and ^1^H-^13^C HSQC) were recorded using a Bruker-300, 300 MHz for ^1^H and 75 MHz for ^13^C. Chemical shifts were expressed in ppm (*δ*) using TMS (tetramethylsilane) (Aldrich Sigma, Germany) as reference. For NMR analysis, the sample was dissolved in 400 *μ*L of CDCl_3_-MeOD (2 : 1) and transferred to a NMR tube.

## 3. Results and Discussion

Our previous investigations allowed determining that the hydroethanolic extract of* H. floribunda *inhibited growth of* M. ulcerans *with a MIC value of 125 *μ*g/mL using the resazurin microtiter assay (REMA) [3]. The CH_2_Cl_2_ fraction of this extract also inhibited growth of* M. ulcerans *with a MIC value of 125 *μ*g/mL and contained alkaloids. Fractionation of an enriched alkaloid extract (MIC = 62.5 *μ*g/mL) was undertaken as described in the Experimental Section, allowing the identification of four different alkaloids in five different fractions and isolation of one of them with high purity. The structure of the major compound was identified as holadysamine (3-methyl amino pregna5,16-dien-20-ol) by MS and NMR [8]. LC-MS was used to identify alkaloids in the other fractions, named compounds A, B, and C, and gave molecular formula of, respectively, C_22_H_37_ON (MW: 331.29), C_21_H_33_ON (MW: 315.26), and C_23_H_37_ON (MW: 343.29). According to bibliographic data on compounds isolated from* H. floribunda* [7, 8], they could correspond to holaphyllinol (MW: 331.29), holamine, or holaphyllamine (MW: 315.26), N,N-dimethyl holamine, N-dimethyl holaphyllamine or methyl 6 holaphylline (MW: 343.29).

**Figure 3:**
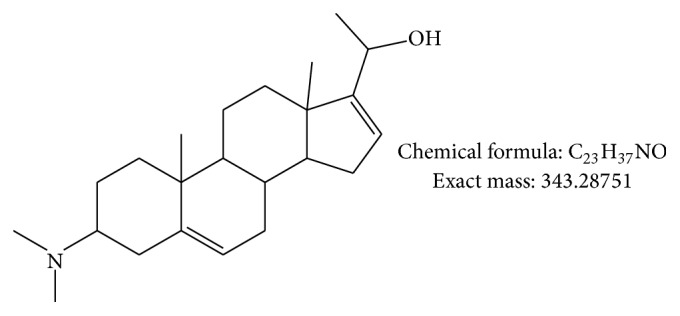
Proposed chemical structure for compound C.

HPLC-ESI-MS/MS allowed us to show that compound A could correspond to holaphyllinol and B to holamine or holaphyllamine while C seems to be a new compound. On the basis of the analysis of spectroscopic data, we observed the presence of a fragment at* m/z* 326 ([M-H_2_O+H]^+^) and the loss of a water molecule in the MS/MS fragmentations of C allowing us to propose an alcoholic and not a ketonic function on C20 (Figure 3).

Further studies must be performed to confirm these chemical structures. It could not be done because of the lack of compound.

Fraction containing pure holadysamine was found to be more active (MIC = 50 *μ*g/mL) than fractions containing A, B, or C or the crude hydroethanolic extract (125 *μ*g/mL) but less active than the reference rifampicin (MIC = 2 *μ*g/mL). This low activity could not totally explain the use of this plant in traditional treatment but as this plant is used in mixtures with other plants; the various compounds present in these plant extracts can act synergistically as it is the case with the antibiotics ethambutol, clarithromycin, and rifampicin [12]. Rastogi and Labrousse in their study showed that the use of these antibiotics in combination results in increased bactericidal effects compared to drugs used alone or in combination with two of them.

## 4. Conclusions

Our results show that extracts of aerial parts of* H. floribunda*, used by traditional healers to treat BU, exhibited significant* in vitro* antimycobacterial activities. Four active alkaloids have been identified. Holadysamine the major compound was found to be the most active one (MIC = 50 *μ*g/mL), but this activity is lower than that of rifampicine. As synergy may be found between different compounds, it would be interesting to analyze the efficacy of the other alkaloids and their combinations. Furthermore, as this plant is used in association with other ones, it is also interesting to test plant associations as used by traditional practitioners. Remedies that would prove effective activity should be applied for toxicological and pharmacological studies. There is also an urgent need of standardization of traditional remedies based on plants.
